# Quantifying antimicrobial use and trends in commercial poultry and dairy farms in Bangladesh

**DOI:** 10.1371/journal.pone.0352962

**Published:** 2026-07-10

**Authors:** Mohammad Eliyas, Kazi Tahmina Karim, Muzaffar Goni Osmani, Sk Shaheenur Islam, Easrat Jahan Esha, Nure Alam Siddiky, Kazi Kamaruddin, Nelima Ibrahim

**Affiliations:** 1 Department of Livestock Services, Dhaka, Bangladesh; 2 Fleming Fund Country Grant, Dhaka, Bangladesh; University of Minnesota, UNITED STATES OF AMERICA

## Abstract

Antimicrobial resistance (AMR) continues to be detected in bacteria from food producing animals in Bangladesh. Globally, as well as in many low- and middle-income countries (LMICs), antimicrobial use (AMU) is identified as the primary driver of rising AMR. This pilot study aimed to quantify AMU in poultry (broiler and layer) and dairy farms in Gazipur and Chattogram districts, assessing seasonal and species-specific AMU patterns. A longitudinal survey conducted during the summer and winter seasons of 2022–2023 involved 36 purposively selected farms (12 each broiler, 12 layer and 12 dairy). AMU was quantified using kilograms of antimicrobial active ingredients (AAIs) and milligrams per population correction unit (mg/PCU). AMU quantity was highest in layer farms (285.80 mg/PCU), followed by broilers (173.29 mg/PCU) and dairy farms (3.56 mg/PCU). Seasonal variations revealed lower AMU levels in broilers (125.68 mg/PCU) and layers (130.48 mg/PCU) in winter, whereas dairy usage increased in winter (13.93 mg/PCU). Predominantly used AAI’s included neomycin (broilers, both seasons), oxytetracycline (layers in summer), tylvalosin (layers in winter), sulfonamides (dairy in summer), and penicillin (dairy in winter). Poultry farms utilised substantially higher AMU quantities. Notable geographic differences in AMU quantities used in broiler and layer farms were observed, while dairy farms showed uniform use across districts. Of important public health concern is the high proportion (65–90%) of antimicrobials that fall under the World Health Organization’s (WHO) Medically Important Antimicrobial (MIA) categories, particularly Critically Important (CIA) or Highest Priority Critically Important Antimicrobials (HP-CIA), as well as the substantial use of Watch- and Reserve-group antimicrobials according to the WHO AWaRe framework. These findings highlight excessive use of medically important antimicrobials and emphasise the urgent need for regulatory action and improved stewardship. This pilot study provides essential baseline data for developing farm-level national AMU surveillance system in Bangladesh.

## Introduction

Antimicrobial resistance (AMR) is an escalating threat to global health, food security and animal production. A key strategy for mitigating AMR is the generation and availability of surveillance data on antimicrobial use (AMU) and antimicrobial consumption (AMC), as emphasised in Bangladesh’s National Antimicrobial Resistance Surveillance Strategy [1]. This national strategy aligns with global frameworks such as the World Health Organization’s (WHO) Global Action Plan on AMR [2], the Food and Agriculture Organization’s (FAO) AMR Action Plan [3] and the World Organisation for Animal Health (WOAH) Strategy on AMR and Prudent Use of Antimicrobials [4].

Globally, the increasing demand for animal-sourced foods is driving the expansion of intensive livestock production systems, where antimicrobials are commonly used not only to treat infectious diseases but also prophylactically and as growth promoters [5]. Data on global AMU indicate that approximately half of all antimicrobials are used in food producing animals [6,7]. A study in 2019 estimated global antimicrobial sales at 93,309 tonnes in 2017, with projections indicating an increase of 11.5% to approximately 104,079 tonnes by 2030 [7–9].

In Bangladesh, poultry and dairy products are essential to food security and supporting livelihoods, yet they are persistently compromised by the spread of AMR. Studies in poultry in Bangladesh have consistently reported high levels of antimicrobial resistance (AMR) in both zoonotic pathogens such as *Salmonella and Campylobacter*, as well as in indicator organisms like *Escherichia coli* [10–12].

In Southeast Asia, the overuse of AMs, coupled with poor biosecurity practices, including inadequate hygiene, infection prevention and control measures (e.g., inadequate vaccination), and suboptimal sanitation (e.g., insufficient cleaning and disinfection, failure to observe downtime and rest period, have been identified as major drivers of AMR [13]. The use of AMs is particularly widespread in commercial livestock production systems in low- and middle-income countries (LMICs), where intensification of food animal production, limited veterinary regulatory oversight, and accessibility to over-the-counter AMs contribute to widespread AMU [14]. This problem is exacerbated in countries like Bangladesh, where antimicrobials are often available as over-the-counter products without veterinary oversight [15–17]. Recent studies have similarly highlighted high AMU levels in commercial broiler production, with substantial use of WHO critically important antimicrobials [18,19]. However, it is increasingly recognised that these patterns are not simply a matter of individual farmer behaviour. Historical and sociological scholarship has demonstrated that antimicrobial use in livestock is shaped by systemic factors: the intensification of production, economic pressures on farmers, supply chains that incentivise routine antimicrobial administration, and the historical legacy of promoting antimicrobials for growth promotion and prophylaxis in the absence of veterinary oversight [20–22]. Thus, addressing AMU requires structural interventions rather than behavioural appeals alone.

The inappropriate use of AMs by farmers, including failure to adhere to recommended dosing regimens and withdrawal periods, can lead to AMR development (e.g., prolonged exposure at low dose or insufficient dose/duration) and antibiotic residues in animal-derived food products, posing significant health risks to consumers [23,24]. A widespread lack of awareness regarding the consequences of indiscriminate and prophylactic AMU has led many animal feed dealers and drug sellers to continue promoting routine AMU, especially in poultry production [23–27].

Although the Government of Bangladesh banned the use of AMs in animal feed through the “Bangladesh Fish Feed and Animal Feed Act 2010” [27], later prohibiting colistin in all forms in 2022 [28] and recently enacting the “Drugs and Cosmetics Act 2023” to regulate the manufacture, import, sale, and quality control of drugs and cosmetics, a significant regulatory gap remains, as there is still no specific regulatory framework to ensure the responsible therapeutic use of AMs in animals [29,30].

In response to the AMR threat, Bangladesh has adopted a One Health approach for AMR surveillance and control. A key component of this initiative is the surveillance of AMU in food-producing animals, particularly in the poultry and dairy sectors. This activity is led by the Department of Livestock Services (DLS), with financial support from development partners, notably the Fleming Fund Country Grant to Bangladesh (FFCGB) and the Food and Agriculture Organization (FAO) of the United Nations.

Although several studies have previously quantified AMU in poultry and dairy farms in Bangladesh, there remains a lack of systematic, farm-level AMU surveillance led by DLS at the national level. To address this gap, DLS, with support from FFCGB, conducted a pilot study to quantify AMU in broiler chicken, layer chicken, and dairy cattle farms across two selected districts. The study also aimed to capture spatial and seasonal variations in AMU patterns and evaluate the applicability of standard measurement approaches, including weight-based metrics and internationally recognised classifications such as the revised World Health Organizations (WHO) List of Medically Important Antimicrobials (MIA) [31] and the AWaRE classification (Access, watch and reserve) [32]. These tools were assessed for their utility in guiding antimicrobial stewardship and informing targeted AMU reduction strategies. This pilot complements existing farm-level research and represents a significant step toward the development of a sustainable field-level AMU surveillance system to support national efforts to contain AMR in Bangladesh.

## Methodology

### Sample collection

**Fig 1:**
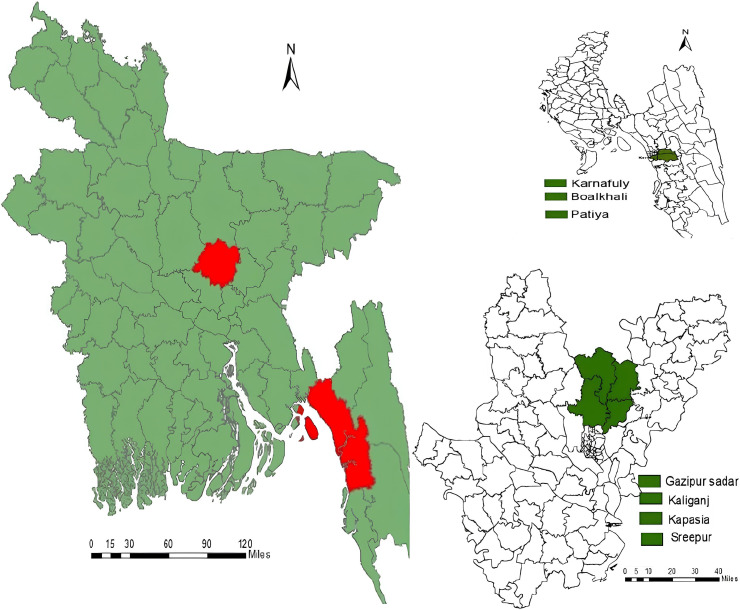
Geo-location of broiler, layer and dairy farms in Chattogram and Gazipur.

A longitudinal study was conducted to quantify AMU in commercial poultry (broiler chickens and layer chickens) and dairy cattle farms across two distinct seasons: the summer/wet season (June–August 2022) and the winter/dry season (December 2022–February 2023). The study was implemented in two high-density livestock-producing districts of Bangladesh, Gazipur and Chattogram, selected in consultation with the respective District Livestock Officers (DLO) who are familiar with the area including the animal production profiles of the two districts (Fig 1).

A total of 36 farms were purposively selected, comprising six broiler, six layer and six dairy farms from each district. For dairy farms, medium-sized (6–25 dairy cattle) and large-sized (>25 dairy cattle) operations, as defined by Heifer International Bangladesh [33], were considered eligible, since smallholder farms typically lack production records. Poultry farms with a flock size exceeding 1,000 birds were included. Only farms with motivated and cooperative farmers who maintained production records were enrolled, and final selection required commitment to participate across both seasons. As farms were selected purposively, findings may not be fully generalisable to all poultry and dairy farms in Bangladesh. The same farms were targeted across both seasons; however, due to participant dropouts, three broiler farms in Gazipur and one broiler and one layer farm in Chattogram were replaced during the winter season. Replacement farms were selected following the same inclusion criteria to maintain consistency.

For broiler farms, AMU was collected for one full production cycle (30–35 days) during each season, while layer and dairy farms were monitored continuously for three months in summer and three months in winter. This approach ensured that AMU was measured consistently across seasons despite differences in production cycles between broilers and continuously operating layer and dairy farms.

Four trained data collectors were recruited, two in each district. Each farm was visited at least once per week. Farmers were trained to collect empty AM sachets, vials, and packages in designated trashcans (garbage bags) and to record AMU data using structured data collection forms provided at the farm level. Newly enrolled farmers during the winter season received hands-on training from the data collectors and DLS resource person on proper sample handling and data recording. Furthermore, basic farm-level demographic data including farm type, flock/herd size, production cycle duration and housing system were recorded at enrolment, as these variables were essential for interpreting AMU patterns.

### Data collection

At the beginning of the study, basic farm-level demographics and flock or herd health management data were collected from each farm by both the data collectors and the supervisor. For routine AMU data collection, farmers stored empty AM packaging materials in trashcans and data collectors compiled the weekly data using standardised AMU recording forms.

Photographs of the packaging materials (both sides) and scanned copies of weekly AMU records were shared via WhatsApp with the DLS resource person for quality control and cross-verification. The DLS resource person and an expert from the FFCGB reviewed the data collection process every 15 days through field visits, to monitor and evaluate implementation of the protocol.

As the DLS is the sole regulatory authority for animal health and routinely conducts epidemiological investigations, separate ethical approval was not applicable. Moreover, no animals were handled or sampled during this pilot study. Farmer participation was voluntary, and informed verbal consent was obtained prior to collecting flock-level data.

### Data analysis

All verified data were compiled and entered into Microsoft Excel by the DLS resource person. Data cleaning and consistency checks were performed before proceeding to further analysis.

**AMU was calculated using the following metrics:**

**a) Antimicrobial active ingredient (AAI):** Formulation of each antimicrobial product such as the AAI(s) and their concentration(s) was taken from the trash can contents or through online search (manufacturer’s website, if available). Product quantities used and active ingredient concentrations were employed to calculate the amount of active ingredient for each antimicrobial (Eq. 1).


$$\begin{array}{c} {AAI}_{kg}=\frac{{Amountofproductused}_{g-ml}\times {Conc.ofantimicrobial}_{\frac{mg}{g-ml}}}{\text{1}\text{0}\text{0}\text{0}\text{0}\text{0}\text{0}} \end{array}$$ (Eq. 1)

**b) Milligrams of active ingredient adjusted for population and weight or population correction unit (mg/PCU):** For poultry, the total amount of active ingredient used in milligrams was divided by the PCU of the flocks on which the respective antimicrobial was administered. PCU was calculated by multiplying the number of birds in respective flocks with 1 kg (standardised average weight of broiler chicken at the time of treatment recommended by the European Surveillance of Veterinary Antimicrobial Consumption (ESVAC)) [34] (Eq. 2, Eq. 3).


$$\begin{array}{c} mg/PCU=\frac{{Totalamountoftheactiveingredientused}_{mg}}{Populationoftreatedflocks\times \text{1}kg} \end{array}$$ (Eq. 2)

For dairy cattle (cows, heifers and calves), the total amount of AAIs used (in milligrams) was divided by the PCU to calculate mg/PCU. PCU was calculated by multiplying the number of animals in each age group with standardised body weights recommended by (ESVAC), i.e., 425 kg for cows, 200 kg for heifers and 140 kg for calves [34] (Eq. 4).

These standardised weights for poultry and dairy were adopted because Bangladesh currently lacks officially established national average body weights at treatment for these categories.


$$\begin{array}{c} {mg/PCU}_{mg/kg}=\frac{{Totalamountoftheactiveingredientused}_{mg}}{Population\times Standardisedweight} \end{array}$$ (Eq. 4)

Cumulative or sum of all flocks or herds, mg/PCU was calculated as:


$$\begin{array}{c} \sum_{n=\text{1}}^{N}{mg/PCU}_{mg/kg}=\frac{\sum_{n=\text{1}}^{N}{\left({Totalamountoftheactiveingredientused}_{mg}\right)}_{N}}{\sum_{n=\text{1}}^{N}{\left(PCU\right)}_{N}} \end{array}$$ (Eq. 3)

Where N is the total number of flocks treated.

**c) Differences in any quantitative measurements between categories:** Difference between season and between districts were determined as follows.


$$\begin{array}{c} \%difference=\frac{\left[Season\text{2}-Season\text{1}\right]}{Season\text{1}}\times \text{1}\text{0}\text{0} \end{array}$$ (Eq. 4)

**d) Absolute change in any quantitative measurements between categories.**


$$\begin{array}{c} Absolutechangeinkg=Season\text{2}-Season\text{1} \end{array}$$

The analysis evaluated AMU based on WHO MIA categories [31], which prioritises antimicrobials according to their importance to human medicine for treating serious human infections and for which limited or no alternatives exist. It helps guide the veterinary sector in restricting or phasing out antimicrobials to preserve their efficacy. The visualisations highlight seasonal variations and species-specific differences.

## Results

### Farm-level demographics

The data collectors gathered AMU data from all 36 farms during both the summer and winter seasons. The population sizes of chickens and cattle included in the study are summarised in Table 1. All farms operated under a conventional open shed housing system.

**Table 1:** Basic-farm-level demographics of the poultry flocks and cattle herds in the study.

Species	Broiler chickens			Layer chickens			Cattle		
Seasons	Summer	Winter	Total	Summer	Winter	Total	Summer	Winter	Total
Total population, n	19,055	26,400	45,455	33,841	38,667	72,508	713	674	1,387
Chattogram, n	9,690	11,500	21,190	13,529	9,185	22,714	285	304	589
Gazipur, n	9,365	14,900	24,265	20,312	29,482	49,740	428	370	798

For broiler farms, AMU data were collected for one full production cycle of 30–35 days during each season. In contrast, for layer and dairy farms, data collection spanned three months during the summer (June–August 2022) and winter (December 2022–February 2023) seasons. None of the participating farms reported using antimicrobials as feed premixes or growth promoters; however, prophylactic use of antimicrobials was observed to be common. The average broiler production cycle duration recorded during the study was 33 days.

### Flock-level AMU exposures (Broiler farm, n=12)

Each of the 12 monitored broiler chicken farms used at least one AAI during data collection. In total, 20 different AAIs were administered for therapeutic or prophylactic purposes, with an overall use of 5.88 kg and ranged from 0.01 kg to 1.4 kg.

#### Total AMU.

The quantity of AAIs measured in mg/PCU in broiler chicken flocks under surveillance, organised according to WHO MIA is shown in Table 2. The overall AMU was 155.98 mg/PCU in Chattogram, 191.20 mg/PCU in Gazipur, with a combined average of 173.29 mg/PCU across both districts.

**Table 2:** Quantity of antimicrobials in broiler chicken farms (n = 12), measured in milligrams per population correction unit, 2022-2023.

WHO MIA	Antimicrobial class	Antimicrobial	Summer season			Winter season		
			CTG	GAZ	Total	CTG	GAZ	Total
HPCIA	Polymyxins	Colistin	0.45	0.66	0.55	70.12	0.63	**29.91**
	Quinolones	Ciprofloxacin	20.02	10.78	15.48	0	4.33	1.85
		Enrofloxacin	15.57	1.07	8.44	0.67	0.10	4.93
		Flumequine	2.79	0.85	1.42	0	0	0
		Levofloxacin	0	0.00	0.42	0	0	0
		Norfloxacin	18.58	0.00	9.45	0	0.4	**19.62**
		Pefloxacin	0	2.72	1.34	0	0.11	**5.36**
CIA	Aminoglycosides	Amikacin	0	0.96	0.47	0	0	0
		Gentamicin	9.08	0	4.62	0	0	0
		Neomycin	0	43.62	21.44	71.16	13.89	**46.73**
	Macrolides	Erythromycin	0	22.30	10.96	0	0	0
		Tilmicosin	0	31.77	15.61	0	0	0
HIA	Amphenicols	Florfenicol	20.85	0	10.60	0	0	0
	Lincosamides	Lincomycin	22.55	0	11.47	0	0	0
	Penicillins	Amoxicillin	0	38.22	18.78	8.33	0.10	4.72
	Tetracyclines	Doxycycline	0	5.77	2.83	9.09	0	**5.21**
		Oxytetracycline	35.83	0	18.22	0	0	0
	Trimethoprim and sulfonamides*	Sulfachloropyridazine	2.99	3.01	3.00	0	0	0
		Sulfadiazine	5.57	24.56	14.90	6.67	0.06	2.84
		Trimethoprim	1.71	4.91	3.29	1.33	0.01	0.57
		**Total mg/PCU**	**156.0**	**191.2**	**173.3**	**167.4**	**19.6**	**121.7**

World Health Organization’s medically important antimicrobial classification (grouped according to authorised used): CIA: critically important antimicrobials; HIA: highly important antimicrobials; HPCIA: highest priority critically important antimicrobials.

CTG – Chattogram district.

GAZ- Gazipur district.

Bolded values in grey-shaded cells during the winter season signify increases of more than 50% relative to the summer season (% change = [winter-summer)/winter x 100).

*Under WHO’s class categories, sulfonamides, dihydrofolate reductase inhibitors and combinations

#### Diversity of AAIs, spatial and seasonal differences.

As shown in Table 2, in the summer season, in total 20 AAI were used. The three most used AAIs in Gazipur were neomycin (43.62 mg/PCU), amoxicillin (38.22 mg/PCU) and tilmicosin (31.77 mg/PCU). In contrast, in Chattogram, the top antimicrobials were oxytetracycline (35.83 mg/PCU), lincomycin (22.55 mg/PCU) and florfenicol (20.85 mg/PCU). In the winter season, 10 AAIs were used. The overall AMU was 140.55 mg/PCU in Chattogram, 105.67 mg/PCU in Gazipur, with a combined average of 125.68 mg/PCU across both districts. The top three AAIs used across both districts in this season were neomycin (46.73 mg/PCU), colistin (29.91 mg/PCU), and norfloxacin (19.62 mg/PCU).

Considerable variation was observed between the two districts in the quantities of specific antimicrobials used. During the summer, neomycin usage was significantly higher in Gazipur, whereas oxytetracycline was more extensively used in Chattogram. In contrast, during the winter, colistin had the highest usage in Chattogram, followed by amoxicillin. Increased total AMU between seasons are highlighted in Table 2 (bolded fonts/grey cells).

Two non-medically important antimicrobials (total amount = 80 grams), amprolium (Chittagong) and toltrazuril (Gazipur), licensed for coccidiosis treatment were also captured in the dataset. These were excluded in all quantitative analyses.

### Flock-level AMU exposures (Layer farms n = 12)

Each of the 12 monitored layer farms used at least one AAI during data collection. In total, 18 different AAIs were administered, amounting to total use of 12.9 kg and ranged from 0.07 kg to 1.87 kg.

#### Total AMU.

The quantity of AAIs measured in mg/PCU in layer flocks under surveillance, organised according to WHO’s MIA is shown in Table 3. During summer, the total AMU was 494.27 mg/PCU in Chattogram and 146.95 mg/PCU in Gazipur, with a combined average of 285.78 mg/PCU across both districts. In winter, the total AMU was 240.22 mg/PCU in Chattogram and 72.58 mg/PCU in Gazipur, with a combined average of 130.48 mg/PCU across both districts.

**Table 3:** Quantity of antimicrobials in layer chicken farms (n = 12), measured in milligrams per population correction unit, 2022-2023.

			Summer season			Winter season		
WHO MIA	Antimicrobial class	Antimicrobial	CTG	GAZ	Total	CTG	GAZ	Total
HPCIA	Polymyxins	Colistin	75.45	3.31	32.15	0	9.22	6.04
	Quinolones	Ciprofloxacin	103.48	0.89	41.90	0	0	0
		Enrofloxacin	0	0.30	0.18	0	6.15	**4.02**
		Levofloxacin	10.20	17.23	14.42	25.52	0.00	8.81
CIA	Aminoglycosides	Neomycin	0	0	0	0	23.05	**15.09**
	Macrolides	Erythromycin	0	16.48	9.89	0	0	0
		Tylosin	20.70	0.00	8.27	37.29	0.00	**12.88**
		Tylvalosin	37.60	11.08	21.68	88.85	0.00	**30.68**
HIA	1st generation cephalosporins	Cephalexin	0	3.32	1.99	0	0	0
	Amphenicols	Florfenicol	0	25.80	15.48	20.97	6.15	11.27
	Lincosamides	Lincomycin	0	29.76	17.86	0.00	0.97	0.64
	Tetracyclines	Chlortetracycline	25.38	0	10.15	0	0	0
		Doxycycline	23.86	1.77	10.60	32.63	0.00	**11.27**
		Oxytetracycline	99.34	1.18	40.42	34.96	12.29	20.12
	Trimethoprim and sulfonamides*	Sulfadiazine	0	0.00	0.00	0	12.29	**8.05**
		Sulfonamides	0	16.69	10.02	0	0	0
		Trimethoprim	0	2.75	1.65	0	2.46	1.61
IA	Aminocyclitol	Spectinomycin	0	16.39	9.84	0	0	0
	Pleuromutilin	Tiamulin	98.26	0.00	39.28	0	0	0
		**Total**	**494.27**	**146.95**	**285.78**	**240.22**	**72.58**	**130.48**

World Health Organization’s medically important antimicrobial classification (grouped according to authorised used): CIA: critically important antimicrobials; HIA: highly important antimicrobials; HPCIA: highest priority critically important antimicrobials.

CTG – Chittagong district.

GAZ- Gazipur district.

Bolded values in grey-shaded cells during the winter season signify increases of more than 50% relative to the summer season (% change = [winter-summer)/winter x 100).

*Under WHO’s class categories, these include sulfonamides, dihydrofolate reductase inhibitors and combinations.

#### Diversity of AAIs, spatial and seasonal differences.

During the summer, a total of 17 AAIs were used. In Gazipur, the highest use was observed for lincomycin (29.76 mg/PCU), florfenicol (25.80 mg/PCU) and levofloxacin (17.23 mg/PCU). In contrast, Chattogram recorded substantially higher usage of ciprofloxacin (103.48 mg/PCU), oxytetracycline (99.34 mg/PCU) and tiamulin (98.26 mg/PCU). In the winter, 12 antimicrobial agents were used across the surveyed layer farms (Table 3). The top three antimicrobials used during winter were tylvalosin (30.68 mg/PCU), oxytetracycline (20.12 mg/PCU), and neomycin (15.09 mg/PCU). In Chattogram, the most used agents were tylvalosin tartrate (88.85 mg/PCU), tylosin tartrate (37.29 mg/PCU) and oxytetracycline (34.96 mg/PCU). In Gazipur, the leading antimicrobials were neomycin (23.05 mg/PCU), oxytetracycline (12.29 mg/PCU) and colistin (9.22 mg/PCU).

Substantial differences were observed between the two districts in the quantities of specific antimicrobials used. In the summer, ciprofloxacin was the most commonly used antimicrobial in Chattogram, followed by oxytetracycline and tiamulin, while lincomycin was used more frequently in Gazipur. In contrast, during the winter, tylvalosin had the highest usage in Chattogram and was not used at all in Gazipur, where neomycin was the most used antimicrobial. Increased total AMU between seasons are highlighted in Table 3 (bolded fonts/grey cells).

### Herd-level AMU exposures (Dairy cattle farms n = 12)

Each of the 12 monitored dairy farms used at least one AAI during data collection. In total, 18 different AAIs were administered, amounting to an overall use of 4.9 kg and ranged from 0.0004 kg to 1.6 kg.

#### Total AMU.

The quantity of AAIs measured in mg/PCU in cattle herds under surveillance, organised according to WHO’s MIA is shown in Table 4. The total AMU was 3.6 mg/PCU during summer and 13.9 mg/PCU in winter season.

**Table 4:** Quantity of antimicrobials in dairy farms (n = 12), measured in milligrams per population correction unit, 2022-2023.

WHO'S MIA	Antimicrobial class	Antimicrobial	Summer season			Winter season		
			CTG	GAZ	Total	CTG	GAZ	Total
HPCIA	3rd generation cephalosporins	Ceftiofur	0.02	0.0	0.02	0.0	0.0	0.0
		Ceftriaxone	0.0	0.05	0.05	0.47	0.33	**0.40**
	Quinolones	Ciprofloxacin	0.03	0.00	0.03	0.05	0.00	0.03
CIA	Aminoglycosides	Gentamicin	0.0	0.06	0.06	0.91	0.32	**0.64**
		Neomycin	0.0	0.01	0.01	0.0	0.0	0.0
		Streptomycin	0.26	0.35	0.61	7.45	2.54	5.18
HIA	Penicillins	Amoxicillin	0.08	0.08	0.16	0.61	0.0	**0.33**
		Ampicillin	0.07	0.07	0.14	1.78	0.13	**1.02**
		Penicillin	0.26	0.37	0.63	6.72	5.75	**6.27**
	Tetracyclines	Oxytetracycline	0.01	0.03	0.04	0.12	0.0	**0.06**
	Trimethoprim and sulfonamides*	Sulfonamides	0.01	0.0	0.01	0.0	0.0	0.0
		Trimethoprim	0.75	1.03	1.78	0.0	0.0	0.0
IA	Bacitracins	Bacitracin	0.01	0.01	0.02	0.0	0.0	0.0
		**Total**	**1.50**	**2.06**	**3.56**	**18.11**	**9.07**	**13.93**

World Health Organization’s medically important antimicrobial classification (grouped according to authorised used): CIA: critically important antimicrobials; HIA: highly important antimicrobials; HPCIA: highest priority critically important antimicrobials.

CTG – Chittagong district.

GAZ- Gazipur district.

Bolded values in grey-shaded cells during the winter season signify increases of more than 50% relative to the summer season (% change = [winter-summer)/winter x 100).

*Under WHO’s class categories, these include sulfonamides, dihydrofolate reductase inhibitors and combinations.

#### Diversity of AAIs, spatial and seasonal differences in AMU.

During the summer, a total of 13 AAIs were documented. Penicillin and streptomycin were the two most used antimicrobials in both Chattogram and Gazipur (Table 4). Commercially available combined antimicrobials containing both penicillin and streptomycin were the most frequently used antibiotics found in the surveyed dairy farms (Table 4). Overall mg/PCU was substantially higher in Gazipur (2.06 mg/PCU) compared to Chattogram (1.50 mg/PCU).

Table 4 showed, the total combined AMU was 13.93 mg/PCU with Chattogram being higher (18.11 mg/PCU) compared to Gazipur (9.07 mg/PCU). Among the top two antimicrobial drugs used during the winter, streptomycin was used at the highest level (7.45 mg/PCU) followed by penicillin (6.72 mg/PCU) in Chattogram whereas, in Gazipur the use was highest for penicillin (5.75 mg/PCU) followed by streptomycin (2.54 mg/PCU).

### Multi-species integration: the value of reporting AMU data using different metrics and antimicrobial classifications to inform stewardship

#### Relative contribution of antimicrobial classes across animal species.

**Fig 2:**
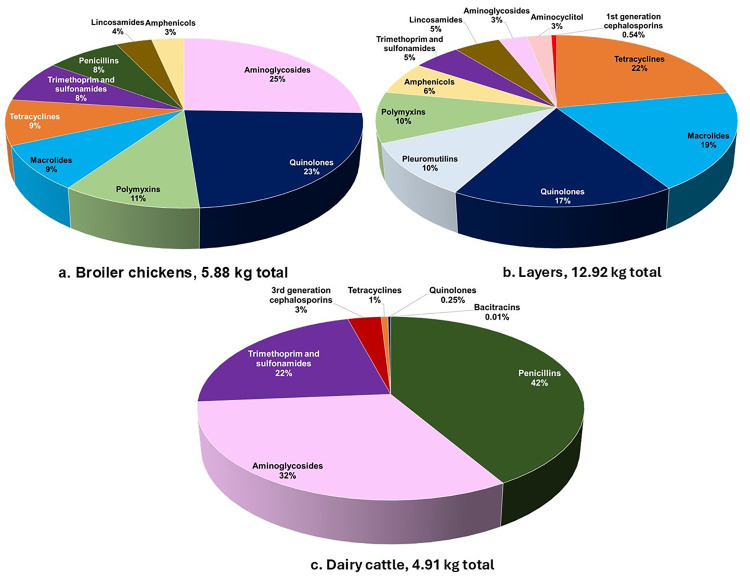
Multispecies overview: the relative contribution of antimicrobial classes used in broiler chicken and layer flocks and dairy cattle herds measured in kg active ingredients, 2022-2023.

Fig 2 presents a summary of the individual AAIs listed in Table 2, aggregated by antimicrobial class across the three animal species under surveillance. This visualisation illustrates the relative contribution of each antimicrobial class to the overall use. In broiler chickens, a total of 5.88 kg of antimicrobials, comprising 3.32 kg in the summer and 2.56 kg in the winter, was used across the 12 farms surveyed, representing 9 distinct antimicrobial classes. The aminoglycosides, quinolones and polymyxins contributed 68% of the total AMU quantity. In layer chickens, a total of 12.92 kg (9.67 kg summer; 3.25 kg winter) of antimicrobials belonging to 11 antimicrobial classes was used. The top classes used varied compared to broiler chickens where tetracyclines, macrolides, quinolones, and pleuromutilins contributed to 69% of the total AMU quantity. In dairy cattle, a total of 4.91 kg (2.14 kg summer; 2.77 kg winter) antimicrobials belonging to 6 distinct classes was used.

#### Patterns of use according to WHO’s medically important antimicrobial classification.

In broiler chickens, a notable increase in the use of HPCIAs was observed between summer (37 mg/PCU; 21% of total summer AMU) and winter (62 mg/PCU; 51% of total winter AMU). In contrast, the use of CIAs remained relatively stable, at 53 mg/PCU (31%) in summer and 47 mg/PCU (38%) in winter. The use of HIAs showed a substantial decline, from 83 mg/PCU (48%) in summer to 13 mg/PCU (11%) in winter. In layer farms, HPCIA and CIA use during summer were equivalent (each 4 mg/PCU, representing 21% of seasonal AMU). However, winter data revealed a disproportionate increase in CIA use to 45%, while HPCIA use showed a modest decline to 14%. HIA use in layers remained relatively consistent across seasons, accounting for 47% of AMU in summer and 41% in winter. Notably, a subset of the IAs category, comprising aminocyclitols and pleuromutilins, was identified during summer, contributing 2 mg/PCU (11%) to total AMU, as previously detailed in Table 3.

**Fig 3:**
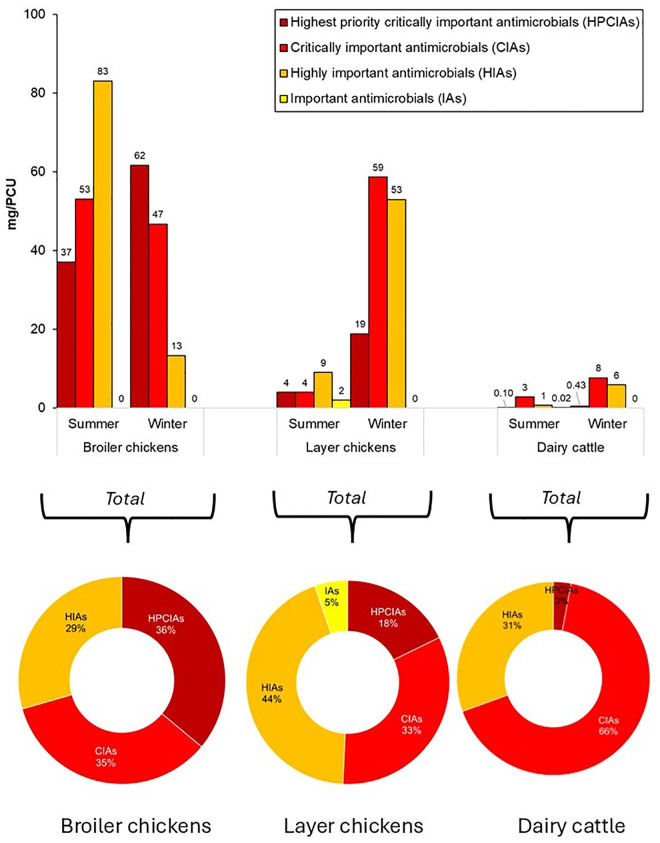
WHO’s medically important antimicrobial classification multispecies overview: quantity of antimicrobials used in broiler chicken and layer flocks and dairy cattle herds measured in milligrams per population correction unit, 2022-2023.

In dairy cattle, AMU was markedly lower than in the two poultry commodities. HPCIA use was minimal in both seasons (<1 mg/PCU; 3% of total AMU). CIAs constituted the largest share of AMU in dairy cattle, accounting for 3 mg/PCU (78%) in summer and 6 mg/PCU (43%) in winter (Fig 3).

When aggregated across species and seasons, HPCIAs were predominantly used in broiler (36%) and layer (18%) chickens, with minimal usage detected in cattle. Across all species, CIAs and HIAs consistently represented the largest proportion of AMU, underscoring their continued importance in food animal medicine in Bangladesh.

#### Patterns of use according to WHO’s AWaRE classification.

The WHO’s AWaRe classification (Access–Green, Watch–Yellow, Reserve–Red), originally developed for human health and utilised in WHO GLASS AMU reports or interactive data visualisation [35], was applied to the current AMU dataset to complement the WHO MIA categorisation. Incorporating the AWaRe classification system into our animal AMU data offers an alternate approach for harmonised and integrated AMU surveillance across the human and animal health sectors under a One Health framework. In broiler chickens, antimicrobials from the Reserve group, primarily colistin, a polymyxin, represented a small but seasonally variable proportion of total AMU, increasing from 1 mg/PCU in summer to 30 mg/PCU in winter. Watch category antimicrobials were the most frequently used in broilers across both seasons, accounting for 101 mg/PCU (58% of total AMU) in summer and 78 mg/PCU (61%) in winter. In contrast, Reserve antimicrobial use in layer chickens showed an opposite trend: colistin was used more extensively in summer (32 mg/PCU) than in winter (6 mg/PCU). The Watch category remained predominant in layers in both seasons, contributing 114 mg/PCU (46% of total AMU) in summer and 68 mg/PCU (52%) in winter. Access category antimicrobials were also widely used in layers chickens, with quantities comparable to the Watch group (100 mg/PCU in summer and 56 mg/PCU in winter). A notable proportion of AMU in layer chickens was attributable to uncategorised antimicrobials in the AWaRe classification, particularly veterinary pleuromutilins (e.g., tiamulin), recorded at 39 mg/PCU in summer. In dairy cattle, antimicrobials were limited to the Access and Watch categories, with Access classes comprising the majority: 3 mg/PCU in summer and 8 mg/PCU in winter. Watch category use was comparatively lower (1 mg/PCU in summer, 6 mg/PCU in winter).

**Fig 4:**
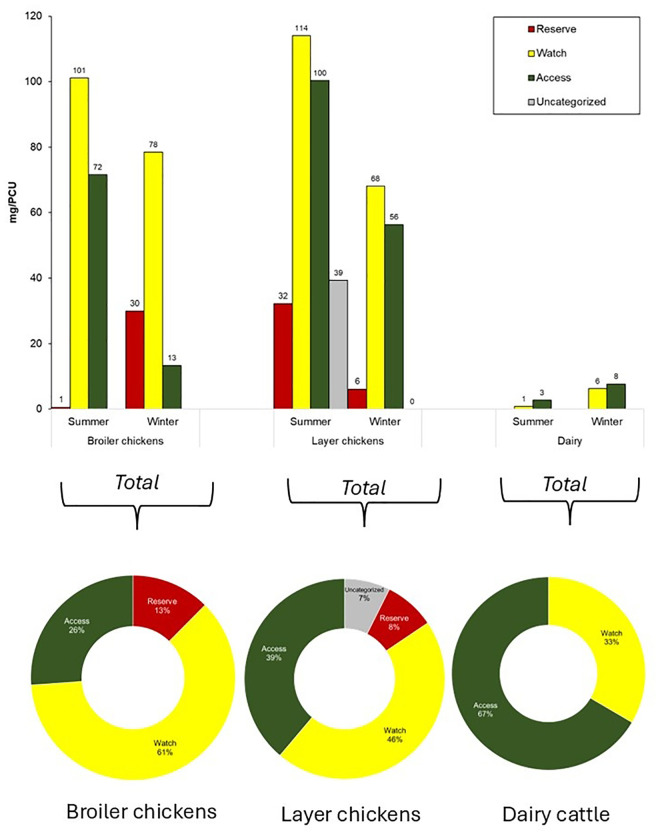
WHO’s AWaRE classification of antimicrobials multispecies overview: quantity of antimicrobials used in broiler chickens and layer flocks and dairy cattle herds measured in milligrams per population correction unit, 2022-2023.

Overall, the Watch category was the most prevalent across poultry species, accounting for 61% of total AMU in broiler chickens and 39% in layer chickens. In contrast, Access antimicrobials dominated in dairy cattle, representing 67% of total AMU (Fig 4). These findings underscore the potential value of AWaRe classification in veterinary AMU monitoring to support targeted stewardship and regulatory action.

## Discussion

Surveillance of AMU in food-producing animals is a core objective of the WHO Global Action Plan on AMR [2]. In several European Union countries, the establishment of national and farm-level AMU surveillance programs has led to significant reductions in antimicrobial use within food animal production systems [36]. Although several studies in Bangladesh have previously assessed or quantified AMU in poultry and dairy farms, the current study provides valuable, field-based quantitative data to support the country’s piloting efforts in AMR surveillance. Uniquely, this study was conducted directly by DLS, with support from the FFCGB. In addition to quantifying AMU, it also explored trends and highlighted spatial and seasonal variations, explored the utility of international AMU classification systems, offering critical insights for future integrated analysis (multispecies, between humans and animals) and inform the progressive enhancements of antimicrobial stewardship strategies.

In this study, AMU was measured using internationally recognised weight-based metrics, including the amount of AAIs in kilograms and mg/PCU, ensuring comparability with global surveillance frameworks and research studies. EMA standard poultry weights were used for calculating the PCU to maintain alignment with international methodology, as nationally established reference weights are currently unavailable in Bangladesh. For dairy cattle, actual average body weights (supplementary table 1 and 2) recorded at the time of treatment were 332 kg for cows, 168 kg for heifers, and 80 kg for calves were incorporated into the analysis. These values are deemed lower than the ESVAC-recommended standardised weights of 425 kg, 200 kg, and 140 kg, respectively [34]. This discrepancy represents a limitation of the study. These lower body weights can be explained by the characteristics of Bangladeshi crossbred dairy cattle, which differ substantially from the relatively homogeneous purebred cattle found in many European countries. In Bangladesh, dairy animals are typically crosses between small-framed indigenous breeds and Holstein Friesians, resulting in smaller average body sizes and variable performance [31,32]. This variation is primarily due to genetic heterogeneity across generations, as well as differences in nutrition and management practices [37,38]. While PCU is a useful indicator for harmonised reporting, it has recognised limitations at farm level. Importantly, mg/PCU treats all antimicrobial active ingredients as equivalent by mass, irrespective of marked differences in therapeutic dosage, potency, or pharmacokinetic properties across drug classes. For example, macrolides are typically dosed at 10–20 mg/kg while tetracyclines may require 30–50 mg/kg, meaning that comparisons of mg/PCU across classes do not reflect true clinical exposure or resistance selection pressure. Alternative indicators such as animal level of exposure to antimicrobials (ALEA), antimicrobial consumption factor, Treatment Incidence or used daily dose (UDD) or animal daily dose (ADD) could not be calculated because continuous liveweight and dose-specific data were not available for poultry in this pilot. Furthermore, although the same farms were intended to be followed across seasons five poultry farms withdrew and were replaced. These substitutions may introduce bias because the replacement farms might differ in disease patterns or AMU behavior.

The AMU values (173.29 mg/PCU) reported for broiler chickens in this study are higher than those reported in previous studies from high-income countries. For instance, Canadian broiler farms reported average AMU ranging from 98 to 134 mg/PCU [39,40] but lower than commercial broiler production in Pakistan, which reported 462.5 mg/PCU) [41]. The comparatively elevated AMU in Bangladeshi broiler farms may reflect several factors, including limited regulatory enforcement, suboptimal biosecurity practices, high disease burden, and the routine use of antimicrobials for prophylaxis in the absence of adequate veterinary oversight [42,43].

For layer chickens, our estimates (125.68–130.48 mg/PCU) fall within the range observed in LMICs, although published data are limited. A study from the Mekong Delta in Vietnam reported approximately 870 mg per bird annually, which may translate to higher PCU values depending on production system [44]. Dairy cattle AMU in our study (3.68 mg/PCU in summer and 13.97 mg/PCU in winter) is higher than figures from Canada, where AMU dropped from 1.7 mg/PCU to 0.4 mg/PCU following strengthened regulations [45] but remains well below the global average of ~45 mg/PCU reported for cattle [46]. Variations in production systems and differences in calculation methodologies make it challenging to directly compare AMU data across countries and regions. In Europe, most countries report antimicrobial sales data using different national frameworks. Notable examples include ANSES-ANMV (France), BelVet-SAC (Belgium), DANMAP (Denmark), NethMap (Netherlands), SWEDRES-SVARM (Sweden), and UK-VARSS (United Kingdom) [47–52]. Additionally, the ESVAC publishes harmonised annual sales data across EU member states [35]. Antimicrobials sales data of Bangladesh is 38.34 mg per kg of animal biomass [53], which is below the EU‑27 countries average but still higher than the minimum levels seen in the lowest‑usage EU countries [35].

All surveyed broiler farms routinely administered prophylactic antimicrobial courses at various stages of the production cycle, with common use during the first week of life [42,54]. Antimicrobials were delivered only through drinking water, as the inclusion of antimicrobials in feed has been banned in Bangladesh since 2010 [28]. This widespread prophylactic use reflects entrenched practices that could be significantly reduced through the adoption of improved hygiene, biosecurity and farm management measures.

Beyond these farm-level practices, the AMU patterns observed in this study must be understood within broader systemic constraints. Participating farms operated with limited access to veterinary services, faced economic pressures to maintain production during disease outbreaks, and relied on antimicrobials as a low-cost, readily available management tool. These conditions reflect structural drivers documented in other LMIC livestock systems, including pharmaceutical supply chains that incentivise routine use and a historical legacy of antimicrobials as growth promoters [15–17]. Recognizing these systemic factors is essential for designing effective interventions.

This study demonstrated clear seasonal and regional variations in AMU across poultry and dairy farms in Bangladesh. During the summer, AMU was highest in broiler farms (173.29 mg/PCU), likely due to increased respiratory and enteric disease risks exacerbated by heat stress and humidity. In contrast, winter AMU peaked in dairy farms (13.97 mg/PCU), possibly reflecting confinement-related illnesses and cold-induced stress. The overall higher AMU observed in broiler chickens during the summer in our study contrasts with findings from countries such as Morocco and Pakistan, where higher treatment frequencies were reported during the winter season [41,55]. Layer farms showed relatively stable AMU across seasons. These discrepancies may reflect differences in climatic conditions, disease patterns, management practices, or antimicrobial prescribing behaviour across regions. Together, these findings reinforce the need for species-specific, regionally tailored and season-sensitive antimicrobial stewardship strategies, aligned with local veterinary service structures and farm-level realities [31,46].

A high proportion of antimicrobials used in broiler, layer and dairy farms in this study fell under the WHO's CIA and HP-CIA categories. Specifically, 65% and 70% of the antimicrobials used in broilers during summer and winter, respectively, were classified as WHO-CIA or HP-CIA. In layer chickens, CIA use was even more pronounced, with over 94% of antimicrobials used in summer and 84% in winter belonging to these critically important classes. Among dairy farms, 75% (summer) and 88% (winter) of antimicrobials used were categorised as CIA/HPCIA. These percentages are comparable to those reported in Belgium (61%) [56], Thailand (63%) [57] and the EU average (76%) [54], but considerably higher than in Vietnam (36.4%) [58]. The high reliance on critically important antimicrobial classes, particularly in poultry, highlights an urgent need for stricter regulation and targeted stewardship programs in veterinary practice in Bangladesh.

At the time this study was conducted, all forms of colistin (belonging to polymyxins, WHO’S HPCIA and WHO’S Reserve antimicrobial), had been officially banned since 2022 [30,59], and combinations containing ciprofloxacin, a quinolone antimicrobial, were banned in 2019 by the Directorate General of Drug Administration [60]. Despite these regulatory actions, colistin continued to be used on farms, likely due to the remaining availability of stock in the market. However, its use is expected to decline with reduced supply. In contrast, HPCIA’s and Watch, particularly quinolones, remain widely used in poultry production as evidenced in our study.

To prioritise regulatory actions and guide antimicrobial stewardship, analysing AMU class (in addition to individual AAI’s), as well as through the WHO MIA and AWaRe classifications provides a complementary framework for monitoring the impact of interventions aimed at reducing AMU in Bangladesh. These classifications enable more targeted risk assessments and can be analysed alongside AMR trends, supporting Bangladesh’s transition towards integrated AMU-AMR analysis and reporting.

This pilot study was done only on 36 broiler chicken, layer chicken and dairy cattle farms across two districts, covering data from both summer and winter seasons. While the findings provide valuable baseline insights, the study's reliance on a convenience sample limits its representativeness and generalisability to the broader poultry and dairy sectors in Bangladesh. As a preliminary effort, the study underscores the urgent need for broader farm inclusion and systematic surveillance to enable sustainable AMU monitoring. Moving forward, the development of a robust, nationwide AMU surveillance strategy, alongside stronger regulatory measures, will be essential to mitigate antimicrobial resistance in the country’s livestock sector.

## Conclusion

This pilot study offers important baseline data on AMU in broiler, layer and dairy farms across two districts in Bangladesh. It reveals significant seasonal and regional variations. As a pilot study that is not nationally representative, it highlights the critical need to establish a standardised, nationwide AMU surveillance system. Strengthening regulations and promoting responsible antimicrobial practices are essential to address AMR in the livestock sector.

## Supporting information

S1 Table Recorded bodyweights of lactating cows and calves across participating dairy farms. (DOCX)

S2 Table Individual animal bodyweight data (cows, heifers, calves, bull) from dairy farms in Chattogram (CD) and Gazipur (GD). (DOCX)
