# Supplementary material for: Quantifying antimicrobial use and trends in commercial poultry and dairy farms in Bangladesh
Source: PLoS One. 2026 Jul 10;21(7):e0352962. doi: 10.1371/journal.pone.0352962 (PMC13353939; doi:10.1371/journal.pone.0352962)
Supplement: S1 Table — (DOCX) [file pone.0352962.s001.docx]

**S1 table: Recorded bodyweights of lactating cows and calves across participating dairy farms**

| Farm ID | District | Total No. of Lactating Cow | Average Weight (kg) | Number of Calf | Average Weight (kg) |
| --- | --- | --- | --- | --- | --- |
| CD01 | Chattogram | 16 | 250 | 14 | 35 |
| CD02 | Chattogram | 12 | 300 | 8 | 45 |
| CD03 | Chattogram | 10 | 320 | 6 | 45 |
| CD04 | Chattogram | 24 | 275 | 15 | 55 |
| CD05 | Chattogram | 80 | 275 | 57 | 50 |
| CD06 | Chattogram | 12 | 300 | 7 | 40 |
| GD01 | Gazipur | 22 | 330 | 6 | 45 |
| GD02 | Gazipur | 30 | 320 | 18 | 55 |
| GD03 | Gazipur | 52 | 330 | 21 | 50 |
| GD04 | Gazipur | 42 | 320 | 18 | 55 |
| GD05 | Gazipur | 17 | 315 | 7 | 45 |
| GD06 | Gazipur | 18 | 360 | 8 | 70 |
