# Supplementary material for: Quantifying antimicrobial use and trends in commercial poultry and dairy farms in Bangladesh
Source: PLoS One. 2026 Jul 10;21(7):e0352962. doi: 10.1371/journal.pone.0352962 (PMC13353939; doi:10.1371/journal.pone.0352962)
Supplement: S2 Table — (DOCX) [file pone.0352962.s002.docx]

**S2 table. Individual animal bodyweight data (cows, heifers, calves, bull) from dairy farms in Chattogram (CD) and Gazipur (GD)**

| **Farm ID** | **District** | **Animal ID** | **Type** | **Weight (kg)** |
| --- | --- | --- | --- | --- |
| CD01 | Chattogram | CD01-01 | Cow | 300 |
| CD01 | Chattogram | CD01-02 | Heifer | 200 |
| CD01 | Chattogram | CD01-03 | Cow | 320 |
| CD01 | Chattogram | CD01-04 | Heifer | 180 |
| CD01 | Chattogram | CD01-05 | Cow | 330 |
| CD02 | Chattogram | CD02-01 | Cow | 320 |
| CD02 | Chattogram | CD02-02 | Cow | 290 |
| CD02 | Chattogram | CD02-03 | Bull | 350 |
| CD02 | Chattogram | CD02-04 | Cow | 290 |
| CD02 | Chattogram | CD02-05 | Cow | 300 |
| CD03 | Chattogram | CD03-01 | Cow | 300 |
| CD03 | Chattogram | CD03-02 | Cow | 320 |
| CD03 | Chattogram | CD03-03 | Bull | 320 |
| CD04 | Chattogram | CD04-01 | Cow | 300 |
| CD04 | Chattogram | CD04-02 | Calf | 110 |
| CD04 | Chattogram | CD04-03 | Cow | 330 |
| CD04 | Chattogram | CD04-04 | Cow | 300 |
| CD04 | Chattogram | CD04-05 | Cow | 310 |
| CD04 | Chattogram | CD04-06 | Cow | 290 |
| CD05 | Chattogram | CD05-01 | Cow | 300 |
| CD05 | Chattogram | CD05-02 | Cow | 320 |
| CD05 | Chattogram | CD05-03 | Cow | 320 |
| CD05 | Chattogram | CD05-04 | Cow | 300 |
| CD05 | Chattogram | CD05-05 | Calf | 90 |
| CD05 | Chattogram | CD05-06 | Cow | 280 |
| CD06 | Chattogram | CD06-01 | Cow | 390 |
| CD06 | Chattogram | CD06-02 | Cow | 300 |
| GD01 | Gazipur | GD01-01 | Cow | 400 |
| GD01 | Gazipur | GD01-02 | Cow | 350 |
| GD03 | Gazipur | GD03-01 | Cow | 280 |
| GD03 | Gazipur | GD03-02 | Cow | 320 |
| GD04 | Gazipur | GD04-01 | Cow | 320 |
| GD04 | Gazipur | GD04-02 | Heifer | 180 |
| GD04 | Gazipur | GD04-03 | Calf | 120 |
| GD04 | Gazipur | GD04-03 | Cow | 400 |
| GD05 | Gazipur | GD05-01 | Calf | 70 |
| GD06 | Gazipur | GD06-01 | Cow | 240 |
| GD06 | Gazipur | GD06-02 | Cow | 300 |
